# Characterization of phytohormone and transcriptome profiles during protocorm-like bodies development of *Paphiopedilum*

**DOI:** 10.1186/s12864-021-08087-y

**Published:** 2021-11-08

**Authors:** Beiyi Guo, Songjun Zeng, Yuying Yin, Lin Li, Guohua Ma, Kunlin Wu, Lin Fang

**Affiliations:** 1 Key Laboratory of South China Agricultural Plant Molecular Analysis and Gene Improvement, South China Botanical Garden, Chinese Academy of Sciences, Guangzhou, 510650 China; 2 University of Chinese Academy of Sciences, Beijing, 100049 China; 3 Center of Economic Botany, Core Botanical Gardens, Chinese Academy of Sciences, Guangzhou, 510650 China; 4 Guangdong Provincial Key Laboratory of Applied Botany, South China Botanical Garden, Chinese Academy of Sciences, Guangzhou, 510650 China

**Keywords:** *Paphiopedilum*, Transcriptome analysis, Protocorm-like bodies, Somatic embryo, Organogenesis

## Abstract

**Background:**

*Paphiopedilum*, commonly known as slipper orchid, is an important genus of orchid family with prominent horticultural value. Compared with conventional methods such as tillers and in vitro shoots multiplication, induction and regeneration of protocorm-like bodies (PLBs) is an effective micropropagation method in *Paphiopedilum*. The PLB initiation efficiency varies among species, hybrids and varieties, which leads to only a few *Paphiopedilum* species can be large-scale propagated through PLBs. So far, little is known about the mechanisms behind the initiation and maintenance of PLB in *Paphiopedilum*.

**Results:**

A protocol to induce PLB development from seed-derived protocorms of *Paphiopedilum* SCBG Huihuang90 (*P*. SCBG Prince × *P*. SCBG Miracle) was established*.* The morphological characterization of four key PLB developmental stages showed that significant polarity and cell size gradients were observed within each PLB. The endogenous hormone level was evaluated. The increase in the levels of indoleacetic acid (IAA) and jasmonic acid (JA) accompanying the PLBs differentiation, suggesting auxin and JA levels were correlated with PLB development. Gibberellic acid (GA) decreased to a very low level, indicated that GA inactivation may be necessary for shoot apical meristem (SAM) development.

Comparative transcriptomic profiles of four different developmental stages of *P*. SCBG Huihuang90 PLBs explore key genes involved in PLB development. The numbers of differentially expressed genes (DEGs) in three pairwise comparisons (A vs B, B vs C, C vs D) were 1455, 349, and 3529, respectively. KEGG enrichment analysis revealed that DEGs were implicated in secondary metabolite metabolism and photosynthesis. DEGs related to hormone metabolism and signaling, somatic embryogenesis, shoot development and photosynthesis were discussed in detail.

**Conclusion:**

This study is the first report on PLB development in *Paphiopedilum* using transcriptome sequencing, which provides useful information to understand the mechanisms of PLB development.

**Supplementary Information:**

The online version contains supplementary material available at 10.1186/s12864-021-08087-y.

## Background

*Paphiopedilum* Pfitzer (Orchidaceae) is commonly known as slipper orchid due to the resemblance of its pouch-shaped lip to a lady’s slipper. *Paphiopedilum* is one of the most primordial genera of Orchidaceae, comprising 107 species found so far [1]. Certain species of *Paphiopedilum* have high ornamental value because their flowers are available in unique patterns. *Paphiopedilum spp.* are important horticultural plants and an endangered species. Wild populations of *Paphiopedilum spp.* are critically endangered due to habitat destruction and unsustainable harvest. All wild species of *Paphiopedilum* are listed in the Convention on International Trade in Endangered Species of Wild Fauna and Flora (CITES) Appendix I, and their trade is prohibited [2].

Conventional *Paphiopedilum* orchid propagation through axillary buds division from the mother plant is unproductive and time-consuming. Currently, the general method of rapid propagation of *Paphiopedilum* is asymbiotic seed germination. However, the efficiency of asymbiotic germination is limited by the flowering period and capsule maturity [3, 4]. Fully mature *Paphiopedilum* seeds are usually difficult to germinate [3, 5]. In vitro propagation of *Paphiopedilum* showed that when the mature plant organ of shoots, leaves, or roots were used as explants, the induction of adventitious buds is difficult, and the propagation efficiency is low [6, 7].

Orchids have a unique reproductive system. The seeds are minute and simple, lacking a well-defined endosperm and cotyledon. A small spherical tuber-like structure formed from germinating seeds is defined as a protocorm. PLBs, whose general structure and growth characteristics are similar to those of protocorm, are derived from somatic cells [8, 9]. PLBs are the ideal explants for in vitro propagation because PLBs have the ability to generate secondary PLBs and differentiate into complete plants. Under suitable stimulation, PLBs can form a meristematic zone characterized by small cells and no intercellular spaces (some studies refer to it as SAM). Shoots will differentiate from this meristematic zone [10]. In this process, in addition to the changes in endogenous hormones, many storage products undergo metabolism or transport [9, 11].

In *Paphiopedilum,* it is hard to induce PLBs, which leads to the failure of the popularization of this propagation system. Although in vitro propagation system through PLBs formation of several *Paphiopedilum* orchids, such as *P. hangianum* [12], *P. rothschildianum* [13], and *P. nivrum* [14], had been established, such attempts on many other *Paphiopedilum* orchids failed. PLBs induction ability varies greatly among different orchid species and varieties. PLBs of *P*. SCBG Huihuang90 can be induced and proliferate continuously, so it is an excellent plant material for the study on PLBs initiation and development.

Traditionally, researchers have considered both protocorms and PLBs as the “somatic embryo” of orchids because of the morphological and compositional similarities [9, 15]. However, a comparative transcriptome analysis proposed that PLBs and protocorms are molecularly distinct from zygotic embryos in *Phalaenopsis aphrodite*. Instead, PLB regeneration may be derived from the shoot regeneration process [16]. In general, the molecular mechanisms of PLB regulation remain unclear. Besides, whether PLBs are indeed somatic embryos is also controversial. Many marker genes of somatic embryo or genes involved in the regulation of somatic embryogenesis have been identified, such as *SOMATIC EMBRYOGENESIS LIKE RECEPTOR KINASE* (*SERK*), *BABYBOOM* (*BBM*), *WUSCHEL* (*WUS*), and *CLAVATA* (*CLV*) [17–19]. *SERK* has been proven to play an important role in somatic embryogenesis [18]. *WUS* and *CLV* generally maintain stem cell and cell differentiation in stem meristem [19]. *WUSCHEL RELATED HOMEOBOX* (*WOX*) genes are homologous genes of *WUS*. *WOXs* regulate early embryo patterning [20, 21] and contribute to maintaining the stem cell meristem [22, 23]. *BBM* is an important transcription factor involved in plant embryogenesis and a key regulator of plant cell totipotency, [24]. *BBM* can induce the formation of differentiated somatic cells and somatic embryos by activating signal transduction pathways. Analysis of the expression patterns of these genes is worthwhile to investigate the nature of PLBs.

The role of plant hormones during in vitro propagation has been extensively studied. Many studies suggested auxin was a prerequisite for callus induction from protocorm and subsequent PLB maintenance [25]. Studies indicated that auxin influenced protocorm development. In many orchid species, such as *Spathoglottis plicata* [26, 27], *Oncidium* [28], and *Cymbidium mastersii* [29], exogenous auxin application increased protocorm numbers or influenced protocorm morphology during germination. Previous studies showed that exogenous auxin application promoted an undifferentiated state, but reduction or removal of auxin from the culture media resulted in shoot formation [25]. Other plant hormones such as GA and JA also play a significant role in PLB development [30, 31]. Although numerous studies have proven that plant growth regulators (PGRs) play a significant role in PLB induction and development [8], few studies have been conducted on the changes of endogenous hormone content during *Paphiopedilum* PLB induction and development. Endogenous auxin is synthesized in protocorm and affects the growth and development of protocorm and PLBs. IAA is the most common naturally endogenous auxin [32]. The content of IAA is affected by the synthesis, degradation, and transport of IAA, and it functions through signal transduction pathways. TRYPTOPHAN AMIDOTRANSFERASE OF ARABIDOPSIS (TAA1) and TAA-Related (TAR1) family proteins synthesize IPyA from tryptophan, while YUCCA family proteins catalyze the conversion of IPyA to IAA [33, 34]. DIOXYGENASE OF AUXIN OXIDATION (DAO), which belongs to the 2-oxoglutarate (2OG) Fe(B) oxygenase family, is a key enzyme in the IAA oxidation pathway [35]. Main function of DAO is to catalyze the oxidation of IAA to 2-oxoindole-3-acetic acid (oxIAA) in plants [36]. Free IAA can be polar transported to the site of action by auxin transport carrier and participate in signal regulation. Plant-specific PIN-formed (PIN) proteins directly promote cellular auxin efflux [37–39]. Members of the influx carriers AUXIN RESISTANT1/ LIKE AUXIN RESISTANT (AUX1/LAX) are functional auxin influx carriers and mediate auxin-related developmental events in different organs and tissues [40, 41].

Although plant tissue culture has been widely used to propagate orchids, large-scale propagation for *Paphiopedilum* by tissue culture is still a challenge. In this study, PLBs were successfully induced from seed-derived protocorms of *P.* SCBG Huihuang90. The morphological characteristics and level of major endogenous hormones of PLB at different developmental stages were assessed to further understand the biological process of PLBs initiation and development. Comparative transcriptome analysis during *P.* SCBG Huihuang90 PLB initiation and development provided valuable insights into the gene regulatory programs that characterize the PLB developmental process.

## Results

### Morphological characterization of PLB development

**Fig. 1:**
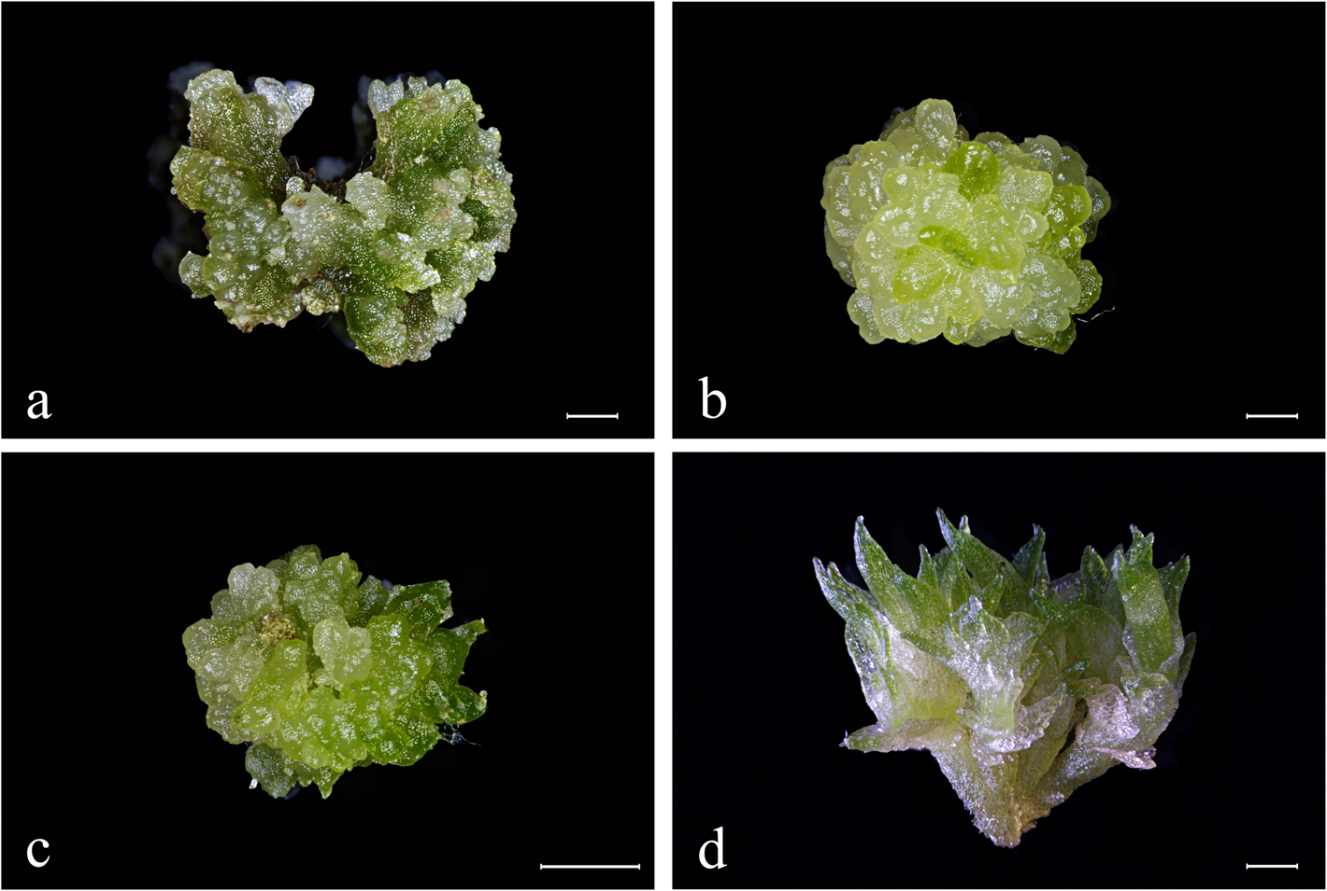
Morphological characterization of the PLBs at four different stages used for RNA-seq analysis. **a** Stage A: Meristem mass. **b** Stage B: newly emerged PLBs. **c** Stage C: Mixture of PLBs and shoots. **d** Stage D: Cluster shoots (Scale bar = 1000 μm)

The growth process of PLBs was divided into four stages based on their morphological characteristics. 2,4-dichlorophenoxyacetic acid (2,4-D) was used to induce protocorms which were derived from germinating of P. SCBG Huihuang90 seeds to form the mass of callus-like meristem (Fig. 1a). The mass of meristem (stage A) was the original material of the PLB induction. At this stage, the inside of the explant was green and part of the edge was light yellow. The surface of the explant was uneven (Fig. 1a). After that, PLBs formed and gradually covered the surface of the mass of meristem (Fig. 1b). It meant that the mass of meristem differentiated into the PLBs mass (stage B). The PLBs mass and the meristem mass were easily distinguished because the PLBs mass had distinct spherical protuberances densely distributed on the outer surface, while the meristem mass appeared lump-like with no fixed shape. The single spherical protrusion was a single PLB, which could eventually develop into a complete plantlet. Next, parts of the PLBs mass differentiated into plantlets (stage C; Fig. 1c). The materials at stage C contained a mixture of shoots and PLBs. Finally, the PLBs mass differentiated into plantlets with green leaves (stage D; Fig. 1d).

**Fig. 2:**
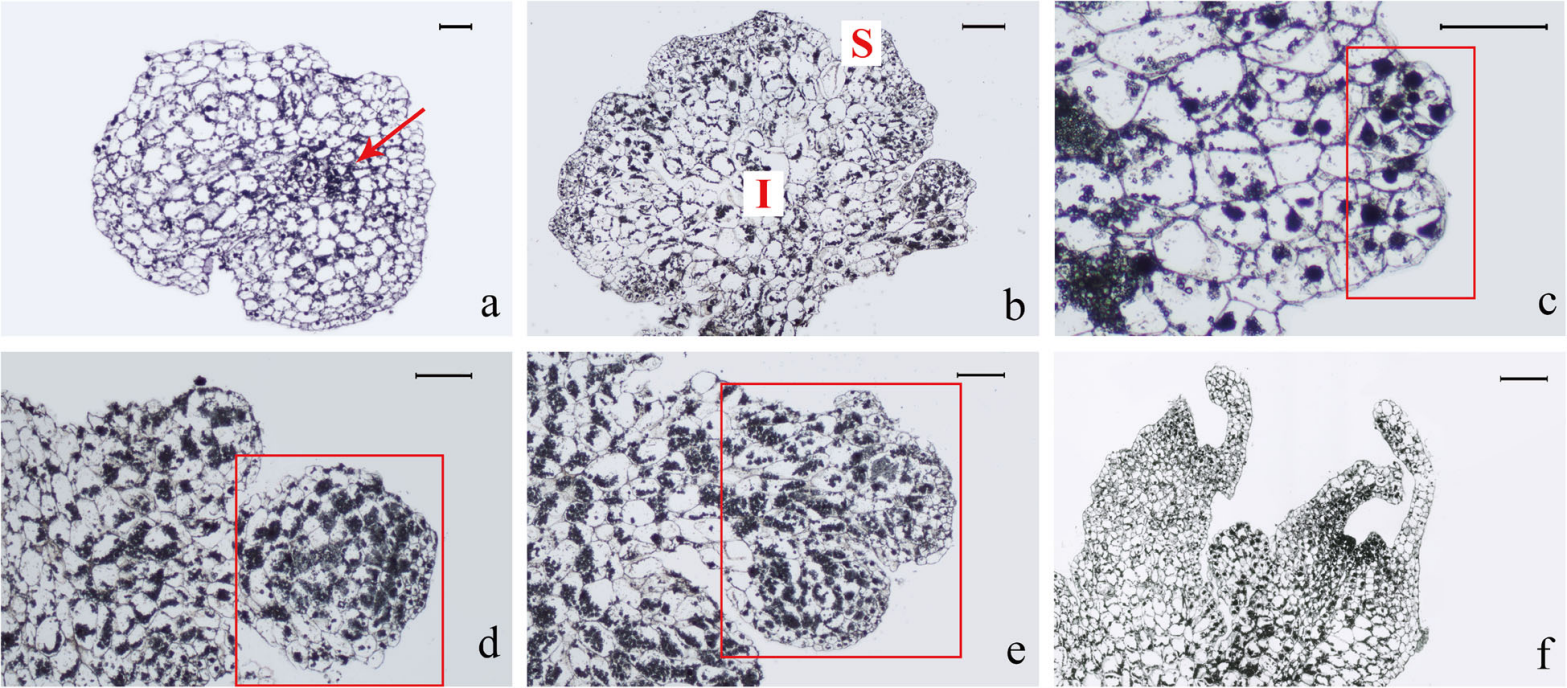
Histological observation of PLB at different developmental stages. **a** Meristem mass. The meristem mass was induced from protocorms by half-strength MS medium containing 0.05 mg/L 2,4-D for 2 months. They composed of a small area of dense, small inner cells (red arrow) and compact aggregates of larger outside cells (Scale bar = 100 μm); **b** PLBs mass. The PLBs mass was induced from meristem mass by half-strength MS medium containing 0.05 mg/L 2,4-D for 2 months. The surface of the newly emerged PLBs mass was covered with small and compact meristem cells (S), which would subsequently develop into shoots. Small protrusions started to form at the surface of the PLB. Inner cells (I) remained larger (Scale bar = 500 μm). **c-e** Local PLBs development process. The compact small cells on the surface continued to divide, resulting in protuberances. They elongated and increased in size, eventually forming spheroids, which were the mature PLBs. The area in the red box depicted the development from a small protuberance to a mature PLB. (c-e. Scale bar = 200 μm); **f** Cluster shoots. The primordial and young leaves differentiated from PLBs (Scale bar = 500 μm)

The microstructure change of PLBs at stages A, B, C, and D were further characterized at the cellular level through histological observations. The meristem mass was mainly composed of aggregates of large cells without intercellular spaces and had compact smaller cell clusters inside (Fig. 2a). These compact small cells might be the center of vigorous division and contribute to the continuous proliferation of the meristem mass by active cell division [13]. As the meristem mass began to differentiate into the PLBs mass, several layers of smaller cells covered on its surface and became tightly packed, while the basal cells remained larger (Fig. 2b). Small protrusions started to form at the surface of newly emerged PLBs mass. Further division of the compact meristem cells, resulting in the elongation and size increase of the protuberances. They eventually formed spheroids, which were the mature PLBs (Fig. 2c, d, e) and shown distinct growth polarity and cell size gradients. The cells at the base of PLB had a different cell fate from those at the apex. The PLB remained polar and continued to elongate (Fig. 2e) until they differentiated into the shoots (Fig. 2f). The primordial and young leaves differentiated from apical cells of PLBs, transforming PLBs into plantlets.

### Change of endogenous hormones during PLB initiation and development

The coordinated interaction of endogenous hormones such as IAA, JA, ABA, trans-zeatin (TZR), and GAs plays multiple important roles in callus and SAM development. The content of several endogenous hormones was determined along with the PLB developmental process.

**Fig. 3:**
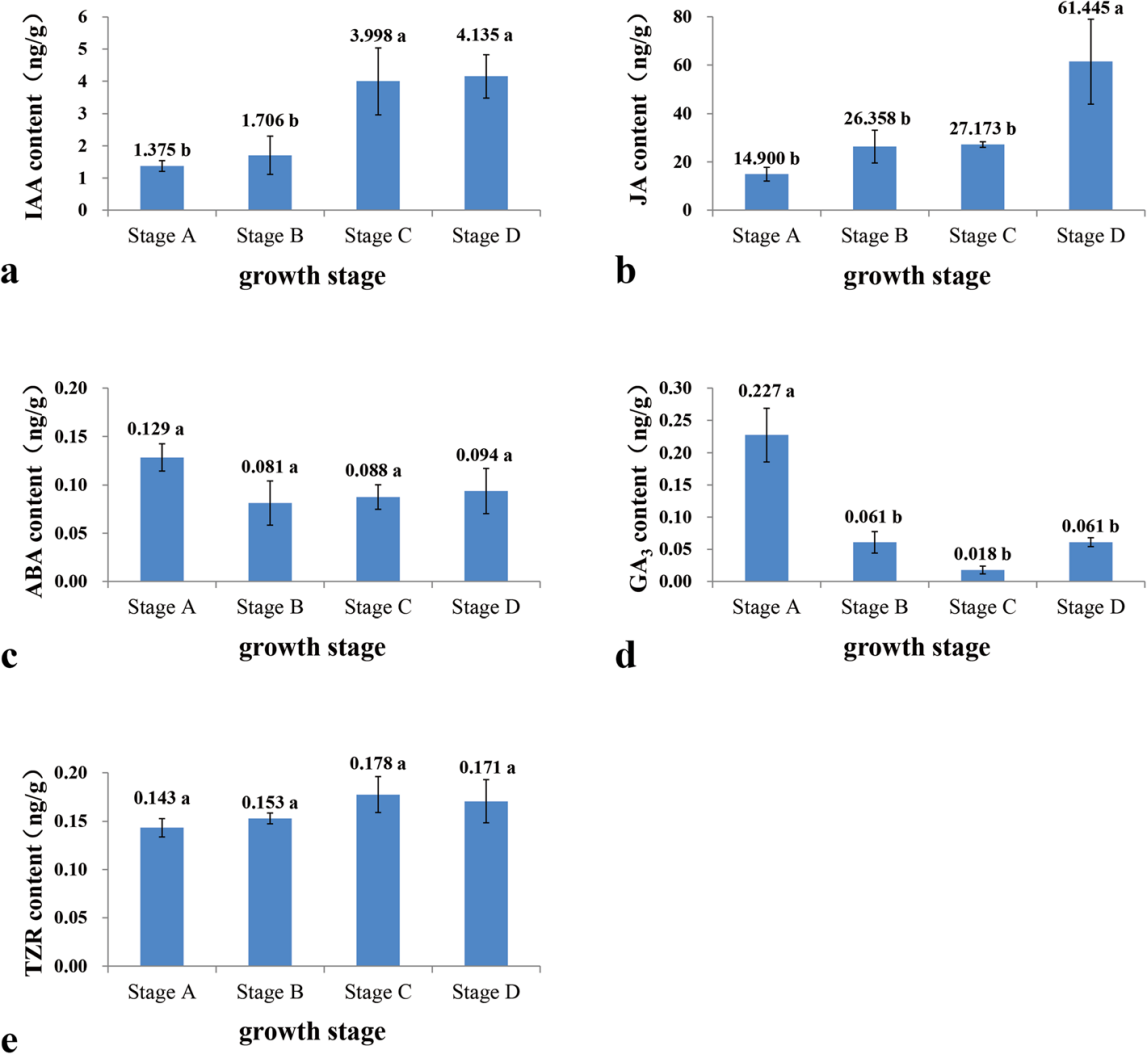
Endogenous IAA (a), JA (b), ABA (c), GA (d), and TZR (e) contents at four different developmental stages of PLBs measured by HPLC. Data represent the average of three biological replicates. Values represent means ± SE (*n* = 3). Bars with different letters are significantly different according to Duncan’s multiple range test at a *P* value < 0.05. Stage A served as control

The content of IAA was significantly lower at stage A and stage B than that at stage C and stage D. The IAA content was 1.38 ng g^− 1^ fresh weight at stage B. The content of IAA at stage C became 1.8 times higher than that at stage A (Fig. 3a). The content of IAA increased with PLBs differentiation until they completely differentiated into shoots. These results suggested that the change in IAA levels may be correlated with PLB development and shoot formation.

The content of GA_3_ decreased to a low level of around 0.06 ng g^− 1^ fresh weight from stage A to stage B (Fig. 3d). This was consistent with previous reports showing that GA_3_ inactivation was required for the activity of the SAM [42–44]. JA content increased sharply during PLBs differentiation into shoots. At stage D, the content of JA was 61.445 ng g^− 1^ fresh weight and significantly higher than other growth stages (Fig. 3b). Previous studies showed that the presence of JA in the medium stimulated the PLB and shoot formation in Hybrid *Cymbidium* [45]. The level of ABA and TZR stayed relatively constant throughout the whole PLB developmental process (Fig. 3c, e).

### De novo assembly and functional annotation

In total, the RNA-seq of 12 cDNA libraries of explants from four different developmental stages (stage A, B, C, and D) produced approximately 80.78 Gb clean reads after removing adapters and filtering low-quality reads and over 10% of unknown nucleotides from raw data. The detailed statistics of clean reads are shown in Table 1. Overall, for all clean reads, the Q30 was over 92.35% and the GC content ranged from 47.53 to 48.80. High-quality sequencing data was de novo assembled and generated 615,869 transcripts with N50 length of 1131 bp. A total of 223,079 longest transcripts were selected as unigenes, with mean length and N50 length of 757.33 and 923, respectively. The detailed results of de novo assembly are shown in Supplemental Table S1.

**Table 1:** Detailed statistics of clean reads

Samples	Read Number	Base Number	GC Content	% ≥ Q30
Stage A-01	18,310,566	5,493,169,800	47.53	92.52
Stage A-02	21,732,828	6,519,848,400	47.73	92.35
Stage A-03	26,813,456	8,044,036,800	48.01	92.89
Stage B-01	23,605,550	7,081,665,000	48.11	93.54
Stage B-02	20,321,782	6,096,534,600	48.12	93.24
Stage B-03	19,516,643	5,854,992,900	47.96	93.71
Stage C-01	20,398,668	6,119,600,400	47.72	93.29
Stage C-02	23,179,966	6,953,989,800	47.94	93.81
Stage C-03	20,303,802	6,091,140,600	48.14	93.14
Stage D-01	22,296,877	6,689,063,100	48.56	93.01
Stage D-02	24,393,463	7,318,038,900	48.62	93.19
Stage D-03	28,401,917	8,520,575,100	48.8	93.01

To determine the functional annotations, software BLAST (http://blast.ncbi.nlm.nih.gov/Blast.cgi) was used to compare the information of sequence or amino acid sequence of unigenes to 7 databases: NR, Swiss-Prot, Kyoto Encyclopedia of Genes and Genomes (KEGG), Clusters of Orthologous Groups (COG), euKaryotic Orthologous Groups (KOG), Gene Ontology (GO), and Protein family (Pfam). Table 2 shows that the total number of annotated unigenes was 105,795. Among them, 19,383 (8.69%), 56,553 (25.35%), 13,733 (6.16%), 58,590 (26.26%), 30,782 (13.8%), 51,766 (23.21%), and 104,149 (46.69%) unigenes were annotated by COG, GO, KEGG, KOG, Pfam, Swiss-Prot, and NR database, respectively.

**Table 2:** Annotated unigenes statistics of different databases

Annotated databases	Number of annotated unigene	Percentage (%)
COG	19,383	8.69
GO	56,553	25.35
KEGG	13,733	6.16
KOG	58,590	26.26
Pfam	30,782	13.80
Swiss-Prot	51,766	23.21
NR	104,149	46.69
All	105,795	47.42

### Differential gene expression analysis and KEGG enrichment

**Fig. 4:**
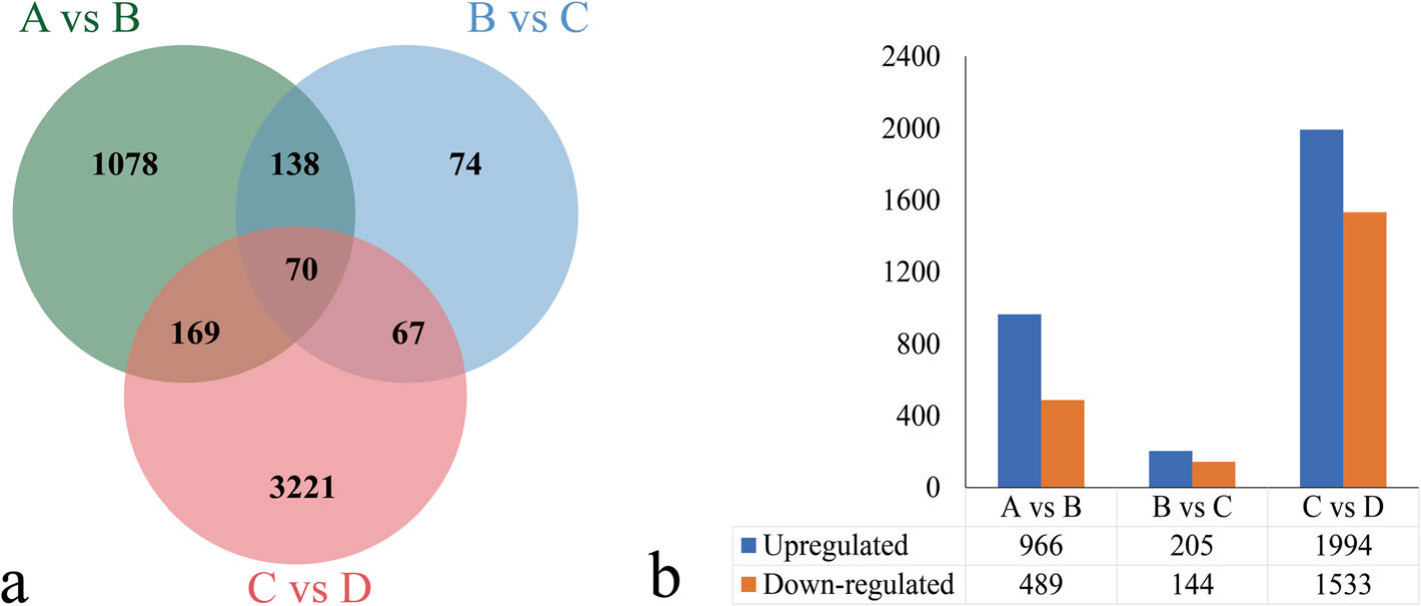
An overview of the number of DEGs. **a** Venn diagram of the number of DEGs in each comparison group. **b** Histogram of the number of upregulated and downregulated DEGs in each comparison group

To understand the mechanism of PLB regeneration at the molecular level, the DEGs between samples were analyzed. To detect DEGs, | log2(Fold Change) | ≥1 and false discovery rate (FDR) < 0.05 were used as screening criteria. The total number of DEGs was 5331 (Fig. 4). In order to understand the differential genes between different periods for analysis, three comparison groups between successive growth stages were designed: stage A vs stage B (A vs B), stage B vs stage C (B vs C), stage C vs stage D (C vs D). The comparison group C vs D contained the largest number of DEGs, with 3527 DEGs, of which 1994 were upregulated and 1533 were downregulated (Fig. 4b). In the comparison group A vs B group, the number of DEGs was 1455, with 966 upregulated and 489 downregulated (Fig. 4b). The comparison group B vs C contained the least number of DEGs, with only 349, of which 205 were upregulated and 144 were downregulated (Fig. 4b). In the three comparison groups, the number of upregulated genes was greater than the number of downregulated genes. There were 70 differential genes shared by the three comparison groups, and there were 1078, 74, and 3221 unique DEGs in group A vs B, B vs C, and C vs D, respectively (Fig. 4a). These specifically expressed genes might regulate distinctive physiological processes during the formation and development of *P*. SCBG Huihuang90 PLBs. A total of 4293 (80.53%) genes were annotated according to at least one database (Table 3). Among the seven databases, in all comparison groups, NR database annotated the most genes (73.75–81.55%), followed by Swiss-Prot (54.85–62.78%).

**Table 3:** Statistics of DEGs annotation in each comparison groups

DEG Set	Swiss-Prot	GO	KEGG	COG	KOG	Pfam	NR	Total
A vs B	682	260	882	1180	293	488	890	1180
B vs C	194	58	230	309	57	92	252	309
C vs D	1538	617	2067	2804	592	1225	2068	2804

KEGG enrichment analysis of DEGs in the comparison group A vs B and B vs C showed that the biosynthesis of secondary metabolites (ko01110) pathway contained the greatest number of DEGs, with 74 and 16, respectively. In the early stage of PLB development, cell division is vigorous, accompanied by multiple substances metabolism. Among the top 10 enriched KEGG pathways in comparison group A vs B, there are four KEGG pathways related to the synthesis of secondary metabolites, including flavonoid biosynthesis (ko00941), phenylpropanoid biosynthesis (ko00940), stilbenoid, diarylheptanoid and gingerol biosynthesis (ko00945), and flavone and flavonol biosynthesis (ko00944) (Table 4). These secondary metabolites may have a significant influence on the early stage of PLB development. In addition, two KEGG pathways are related to amino acid metabolism (ko00350, ko00360) and two are related to energy metabolism (ko00710, ko00910) (Table 4). Among the top 10 pathways of KEGG enrichment in comparison group B vs C, several pathways related to metabolism were uniquely enriched, including isoquinoline alkaloid biosynthesis (ko00950), tropane, piperidine and pyridine alkaloid biosynthesis (ko00960), arginine and proline metabolism (ko00330), and glycerolipid metabolism (ko00561) (Table 4). Moreover, two KEGG pathways are related to amino acid metabolism (ko00350, ko00360) (Table 4). Buds are formed at stage D. In C vs D, DEGs are significantly enriched in photosynthesis (ko00195), photosynthesis - antenna proteins (ko00196) pathway (Table 4).

**Table 4:** List of the top 10 significant enrichment pathways for DEGs

Pathway ID	ko	enrichment factor	q value	gene number
**A VS B**				
Flavonoid biosynthesis	ko00941	9.49	7.55E-11	19
Glycolysis / Gluconeogenesis	ko00010	4.25	6.93E-10	30
Phenylpropanoid biosynthesis	ko00940	4.63	7.51E-09	24
Tyrosine metabolism	ko00350	5.75	2.23E-06	14
Phenylalanine metabolism	ko00360	4.85	2.60E-06	16
Stilbenoid, diarylheptanoid and gingerol biosynthesis	ko00945	9.32	9.45E-05	7
Flavone and flavonol biosynthesis	ko00944	6.28	0.0003917	8
Circadian rhythm - plant	ko04712	4.95	0.0007079	9
Carbon fixation in photosynthetic organisms	ko00710	2.78	0.0017219	16
Nitrogen metabolism	ko00910	3.86	0.0079	8
**B VS C**				
Phenylalanine metabolism	ko00360	10.66	0.0002006	7
Phenylpropanoid biosynthesis	ko00940	5.81	0.0151141	6
Pentose and glucuronate interconversions	ko00040	8.49	0.0173551	4
Isoquinoline alkaloid biosynthesis	ko00950	12.26	0.0209329	3
Tropane, piperidine and pyridine alkaloid biosynthesis	ko00960	10.51	0.0271453	3
Circadian rhythm - plant	ko04712	8.28	0.0456279	3
Tyrosine metabolism	ko00350	6.19	0.0886087	3
Arginine and proline metabolism	ko00330	4.13	0.0993424	4
Glycerolipid metabolism	ko00561	4.94	1	3
Flavone and flavonol biosynthesis	ko00944	7.88	1	2
**C VS D**				
Photosynthesis - antenna proteins	ko00196	12.03	1.14E-16	21
Photosynthesis	ko00195	7.91	3.79E-11	39
Carotenoid biosynthesis	ko00906	3.94	0.0032052	11
Fatty acid elongation	ko00062	4.72	0.0150914	7
Linoleic acid metabolism	ko00591	6.03	0.0235607	5
Pentose and glucuronate interconversions	ko00040	2.64	0.0336898	12
Glyoxylate and dicarboxylate metabolism	ko00630	2.08	0.0304964	19
Cyanoamino acid metabolism	ko00460	2.57	0.0322172	12
Cutin, suberine and wax biosynthesis	ko00073	6.11	0.0402492	4
Alanine, aspartate and glutamate metabolism	ko00250	2.12	0.0397802	16

### DEGs related to IAA metabolism and signaling

**Fig. 5:**
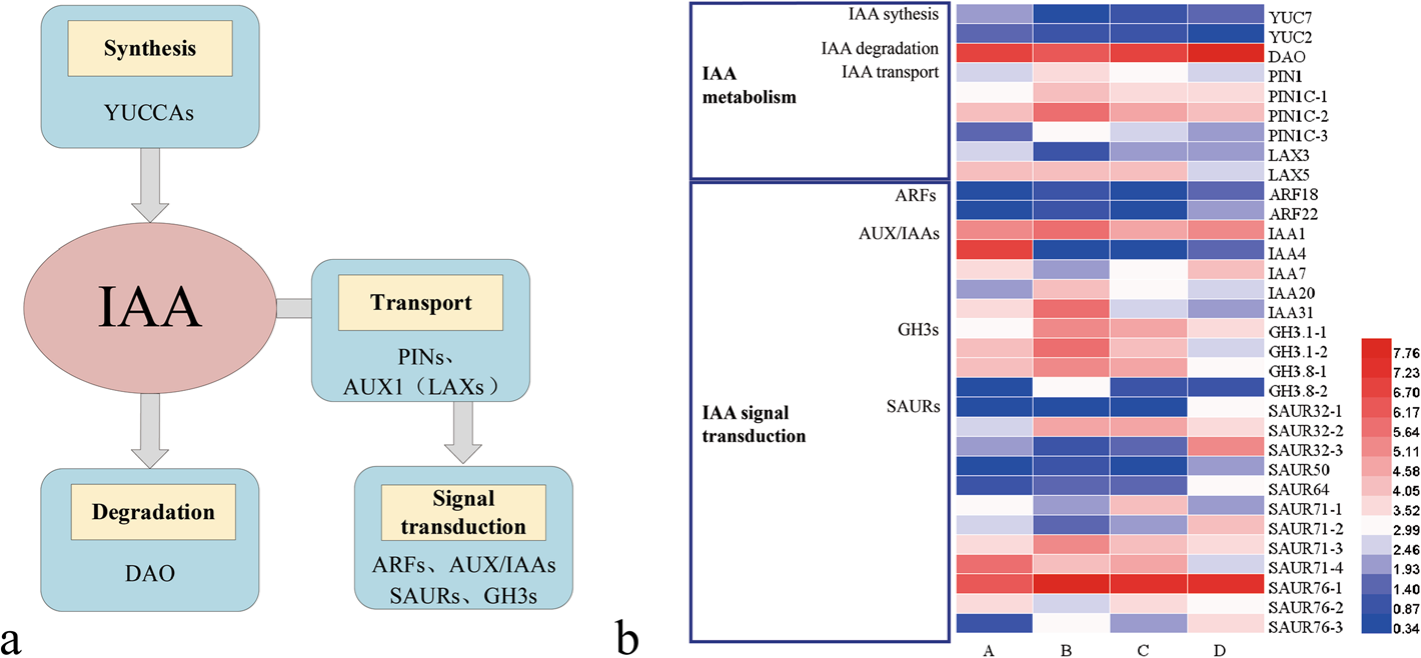
The main pathway and heatmap of DEGs related to IAA. **a** IAA synthesis, transport, degradation, and signal transduction pathways. **b** Heatmap of DEGs related to IAA synthesis, transport, degradation, and signal transduction. The scale bar is shown as the log2 (FPKM+ 1) value. The horizontal coordinates A to D indicate the growth stages A to D

IAA plays an important role in the development of PLBs. A total of 31 DEGs involved in IAA synthesis, degradation, transport, and signal transduction were analyzed (Fig. 5a). In this study, 38 key genes associated with IAA metabolism were identified, including 8 DEGs (Fig. 5b; Supplemental Table S2). Of the 10 *YUCs* that were annotated, only *YUC7* (TRINITY_DN38241_c1_g1) was downregulated at stage B. One *DAO* (TRINITY_DN54506_c1_g1) was upregulated at stage D. The remaining genes related to IAA biosynthesis and deactivation showed no differential expression between the comparisons of adjacent growth stages. Compared with stage A, the expression of *YUC2* (TRINITY_DN56795_c0_g1) and *TAR2* (TRINITY_DN31565_c4_g7) was significantly downregulated at stage D. During the formation and development of PLBs, the expression patterns of these two genes were similar, and both decreased continuously.

A total of 13 *PIN* genes were annotated with different expression patterns (Supplemental Table S2). Four *PIN* genes (TRINITY_DN53433_c1_g1; TRINITY_DN62381_c1_g2; TRINITY_DN66850_c1_g1; TRINITY_DN67065_c2_g2) were significantly upregulated at stage B compared with stage A (Fig. 5b). It can be inferred that these PINs mediated IAA polar transportation during PLB formation. There was no significant difference in the expression level of other *PINs* between successive growth stages.

An *AUX1* and nine *LAXs* were annotated in the transcriptome data. As the PLB developed, the expression level of *AUX1* and most of *LAXs* was stable. Two *LAXs* showed different expression patterns. The expression level of *LAX5* (TRINITY_DN69997_c0_g1) decreased at stage D. The expression level of *LAX1* (TRINITY_DN63572_c1_g2) decreased at stage B (Fig. 5b). Therefore, it can be speculated that the different *LAX* genes may have independent functions at different PLB developmental stages.

According to the plant hormone signal transduction pathway (ko04075) of KEGG, there were mainly five gene families involved in auxin signal transduction, including *TRANSPORT INHIBITOR RESPONSE 1 (TIR1)*, *AUXIN/INDOLE-3ACETIC ACID (Aux/IAA)*, *AUXIN RESPONSE FACTORS (ARFs)*, *Gretchen Hagen3s (GH3s),* and *Small Auxin-Up RNAs (SAURs)*. These auxin responsive genes allow the plants to sense and respond to auxin signals and precisely regulate plant growth and development. During the development of PLBs, 139 genes belonging to these gene families were found to be expressed. Among these genes, there were 23 DEGs in the comparison of A vs B, B vs C, and C vs D (Fig. 5b; Supplemental Table S2). The expression patterns of the three auxin early response factors in the auxin signal transduction pathway are complex because they are all large gene families with functionally redundant members. Compared with the stage A, *IAA20* (TRINITY_DN63682_c1_g2), *IAA31* (TRINITY_DN39077_c0_g1), four *SAUR* genes (TRINITY_DN54779_c0_g1; TRINITY_DN36365_c0_g1; TRINITY_DN42107_c0_g1; TRINITY_DN42107_c1_g1), and four *GH3* genes (TRINITY_DN60352_c0_g1; TRINITY_DN67678_c0_g1; TRINITY_DN67678_c0_g2; TRINITY_DN69497_c0_g1) were significantly upregulated at stage B. On the contrary, *IAA1* (TRINITY_DN63237_c1_g1) and *IAA7* (TRINITY_DN64569_c0_g1) were downregulated. During PLB formation, more auxin early response genes were upregulated than downregulated. In comparison group B vs C, *IAA1* (TRINITY_DN63237_c1_g1), *IAA31* (TRINITY_DN39077_c0_g1), *SAUR50* (TRINITY_DN47796_c0_g1), and *SAUR71* (TRINITY_DN29410_c0_g1) were significantly upregulated while *IAA20* (TRINITY_DN63682_c1_g2) was significantly downregulated. In comparison group C vs D, *IAA4* (TRINITY_DN36308_c0_g1) and five *SAUR* genes (TRINITY_DN62934_c0_g2; TRINITY_DN27590_c0_g1; TRINITY_DN32177_c0_g1; TRINITY_DN36045_c4_g2; TRINITY_DN29019_c0_g1) were significantly upregulated while *GH3.1* (TRINITY_DN67678_c0_g1), *GH3.8* (TRINITY_DN67678_c0_g2) and two *SAUR71* (TRINITY_DN50430_c0_g2; TRINITY_DN29410_c0_g1) were significantly downregulated. For the analysis of auxin early response factors, the number of genes with a significant difference was the least in comparison group B vs C and the most in comparison group A vs B.

### DEGs related to another hormone metabolism and signaling

**Fig. 6:**
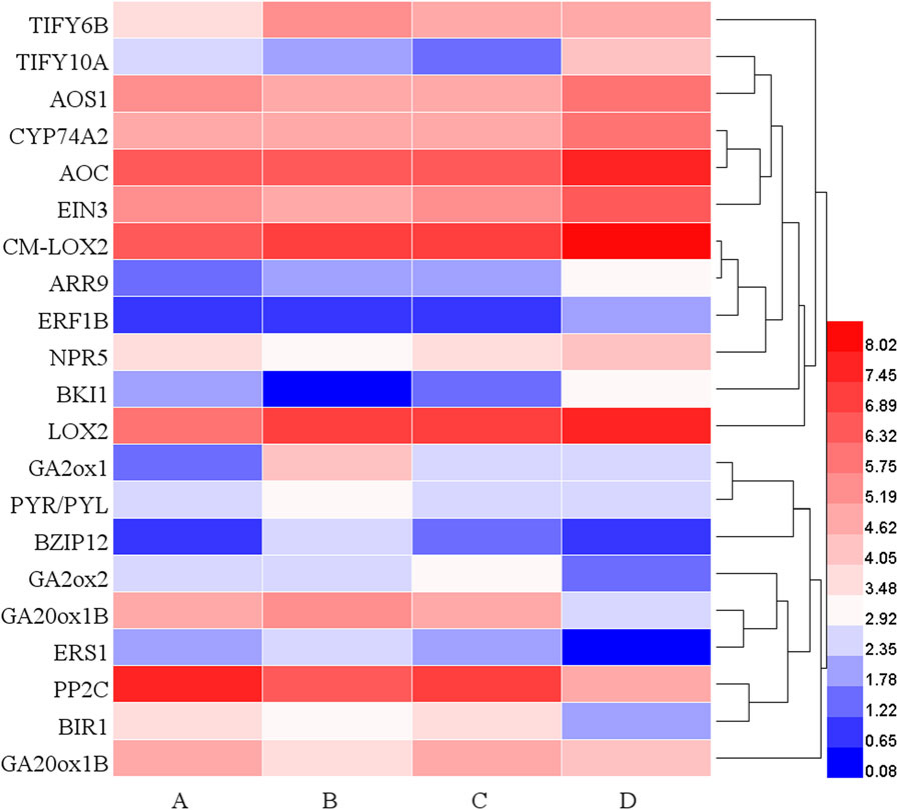
Heatmap of DEGs related to JA, brassinosteroid, ethylene, cytokinine and abscisic acid metabolism, and signal transduction pathway. The scale bar is shown as the log2 (FPKM+ 1) value. The horizontal coordinates A to D indicate the growth stages A to D

We identified four DEGs related to GA metabolism and seven DEGs related to JA metabolism and signaling (Fig. 6; Supplemental Table S3). Gibberellin 20-oxidases (GA20oxs) were major biosynthetic enzymes of some GAs [46, 47]. In our study, two *GA20ox1B* (TRINITY_DN52187_c0_g1; TRINITY_DN42443_c0_g1) genes were downregulated at stage D. *Gibberellin 2-oxidases* (*GA2oxs*) can deactivate bioactive GAs [48]. A *GA2*_*OX*_*1* (TRINITY_DN50547_c1_g1) was significantly upregulated at stage B and a *GA2*_*OX*_*2* (TRINITY_DN41700_c3_g2) was significantly downregulated at stage D (Fig. 6).

Figure 6 showed that a total of five DEGs related to JA synthesis, including two *Lipoxygenase 2* (*LOX2*), *Allene oxide cyclase* (*AOC*), *Allene oxide synthase 1* (*AOS1*) and *Allene oxide synthase 2* (*CYP74A2*) were significantly upregulated at stage D (Supplemental Table S3). Two DEGs involved in the JA signal transduction pathway. *TIFY 6b* (*TIFY6B*) was significantly upregulated at stage B and *TIFY 10a* (*TIFY10A*) was upregulated at stage D.

In addition, ten DEGs involved in brassinosteroid, ethylene, cytokinine, and abscisic acid signal transduction pathway (Fig. 6; Supplemental Table S3). In the comparison group A VS B, *Abscisic acid receptor PYL2* (TRINITY_DN50759_c2_g2) of ABA signal transduction pathway was significantly upregulated and *BRI1 kinase inhibitor 1* (*BKI1*) (TRINITY_DN57604_c0_g1) of brassinostteroid signal transduction pathway was significantly downregulated. In the comparison group B vs C, there were no DEGs related to these plant hormones metabolism and signaling. In the comparison group C vs D, the expression levels of five genes involved in plant hormone signal transduction pathway were upregulated, including *ETHYLENE INSENSITIVE 3* (*EIN3*) (TRINITY_DN69902_c2_g2) and *Ethylene-responsive transcription factor 1B* (*ERF1B*) (TRINITY_DN33907_c0_g1) of ethylene signal transduction pathway, *Two-component response regulator ARR9* (*ARR9*) (TRINITY_DN66618_c1_g1) of zeatin signal transduction pathway, *BRI1 kinase inhibitor 1* (*BKI1*) (TRINITY_DN57604_c0_g1) of brassinostteroid signal transduction pathway, and *ankyrin repeat-containing protein NPR5* (*NPR5*) (TRINITY_DN67985_c1_g1) of salicylic acid signal transduction pathway. On the contrary, *Brassinosteroid LRR receptor kinase BRI1* (*BRI1*) (TRINITY_DN69717_c2_g1) of brassinostteroid signal transduction pathway, *protein phosphatase 2C 3* (*AIP1*) (TRINITY_DN68734_c0_g1) of abscisic acid signal transduction pathway, and *ethylene response sensor 1* (*ERS1*) (TRINITY_DN42513_c0_g1) of ethylene signal transduction pathway were significantly downregulated at stage D.

### DEGs related to somatic embryogenesis

**Fig. 7:**
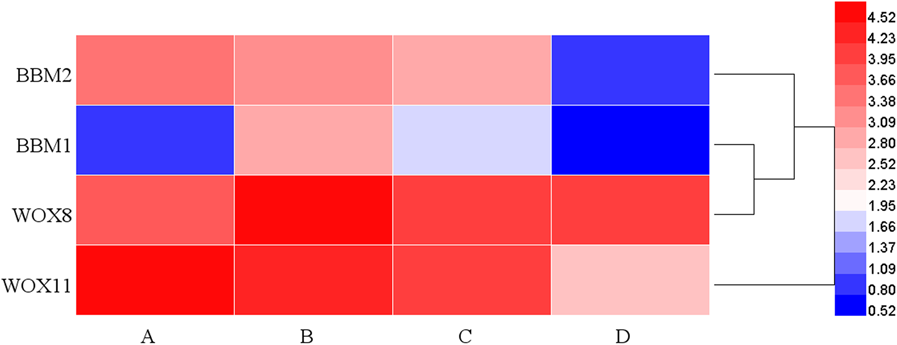
Heatmap of DEGs related to somatic embryogenesis. The scale bar is shown as the log2 (FPKM+ 1) value. The horizontal coordinates A to D indicate the growth stages A to D

In this study, a total of 15 genes related to somatic embryogenesis mentioned above were annotated (Supplemental Table S4). Among these genes, *SERK2*, *BBM2*, two *CLV2*, *WUS*, and six *WOXs* expressed at all growth stages with low expression levels and without specific high expression during PLBs formation and development. In our transcriptome, except for *SERK2*, no other members of *SERK* family were detected. The expression levels of some genes related to somatic embryogenesis were significantly different at different growth stages. Compared with stage A, *WOX8* (TRINITY_DN38477_c0_g1) and *BBM1* (TRINITY_DN51934_c0_g1) were upregulated at the stage B (Fig. 7). Subsequently, the expression of *BBM1* decreased at stage D, while *WOX8* did not. *WOX11* (TRINITY_DN36450_c0_g2) and *BBM2* (TRINITY_DN67396_c0_g1) were downregulated at the stage of shoot formation (Fig. 7). These DEGs may regulate the development and differentiation of PLB.

### DEGs related to photosynthesis

Photosynthesis is a crucial biological process for plant survival. Plants can convert solar energy into organic matter and oxygen through complex light and carbon reactions. Photosystem B (PSII), photosystem I (PSI), light-harvesting complex (LHC), Cytochrome b_6_f (Cyt b_6_f), and adenosine triphosphate (ATP) synthase are key photosynthetic complexes with unique functions [18]. The genes related to the above complex are summarized in two KEGG pathways, photosynthesis (ko00196) and photosynthesis-antenna proteins (ko00195). A total of 36 and 21 differentially expressed genes were detected in photosynthesis-antenna proteins and photosynthesis pathways, respectively (Supplemental Table S5). In comparison group C vs D, most genes related to photosynthesis were upregulated and only two genes were downregulated, all of which were enriched in photosynthesis and photosynthesis-antenna proteins pathway. No genes belonging to photosynthesis and photosynthesis-antenna proteins pathway shown significant differential expression between group B and group C. In comparison group A vs B, two *psaD* (TRINITY_DN66236_c1_g1; TRINITY_DN72036_c3_g2) were downregulated and no genes related to photosynthesis were upregulated.

### Reliability validation of DEG expression via qRT-PCR

**Fig. 8:**
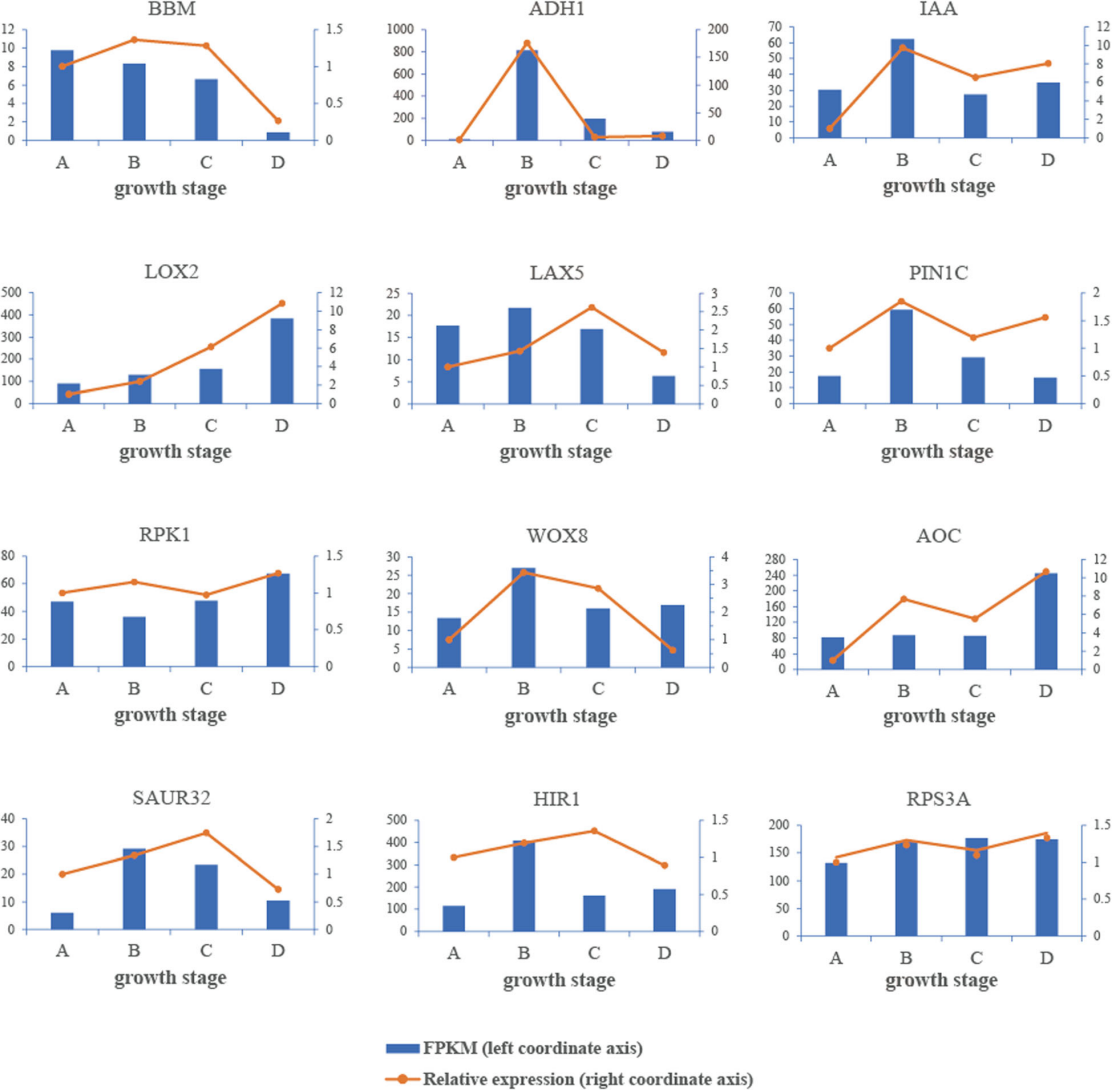
Validation of gene expression in four stages of PLB development by qRT-PCR. Expression levels are normalized to expression levels of *ACT2*. The bar chart and the left coordinate axis represent the expression patterns based on the FPKM value. Line graph and right coordinate axis represent the expression patterns based on the relative expression data of qRT-PCR. Values represent the average of three biological replicates

A total of 12 genes annotated in the transcriptome were selected for quantitative real-time polymerase chain reaction (qRT-PCR) to validate the reliability of the transcriptome sequencing data, most of which were related to plant hormone signal or meristem development. The selected genes had different expressions. For example, both methods validated the high expression of *ADH1* and *IAA* at growth stage B and *LOX2* at growth stage D. The stable expression levels of *RPS3A* and *RPK1* were also verified by both methods (Fig. 8). The trend of the expression pattern obtained by qRT-PCR and RNA-seq of each gene was mostly the same except for a few genes in individual stages.

## Discussions

### Initiation and development of PLBs

In orchids, PLBs can be induced directly from explants or indirectly from callus [49, 50]. So far, it has not been reported that PLBs of *Paphiopedilum* can occur directly from explants. In this study, PLBs were induced from protocorm origin meristem, which is similar to callus, with the ability of continuous proliferation and differentiation. It suggests that the fate of cells may be determined at this stage. Orchids have a unique embryo developmental and germination process. Embryo development arrest at a stage comparable to the globular stage of *Arabidopsis* embryogenesis [10, 51]. As a result, matured embryos are poorly developed without polarized embryo axis establishment. They failed to finish cell fate determination and formation of a body plan. This part is compensated by the presence of protocorm during the late stage of germination. Plentiful DEGs of the comparison group A vs B were enriched in secondary metabolite metabolism, amino acid metabolism, and energy metabolism. Hence, the formation of PLB was accompanied by active energy metabolism as well as multiple substances transport and metabolism (Table 4).

The primary roles of protocorm/PLBs are to establish apical and basal polarity, cell differentiation, tissue specification, and eventually leading to the shoot generation. With the formation of shoots, many genes related to photosynthesis and photosynthesis-antenna proteins were significantly up-regulated. Photosynthesis is essential for the survival and development of plantlets. PLB gradually develops the photosynthetic apparatus and capacity for further autotrophy in the regeneration process. Photosynthetic antenna proteins, which are specialized pigment-protein complexes, allowing for the capture of energy from sunlight, participating in the initial step of photosynthesis [52]. It suggests that PLBs begin to turn green and form leaves at stage D, which enables explants to obtain energy and nutrition through photosynthesis.

Protocorm gives rise to plantlet through the formation of SAM. PLBs have a similar developmental process. The histological observation showed that PLBs of *Paphiopedilum* had a distinct gradient of cell size, with the smaller meristem cells occupying the future shoot pole on the surface and the larger cells located inside (Fig. 2). Similar histological results were also observed in *Phalaenopsis amabilis*. PLBs were composed of smaller compact meristematic cells on the surface of explants at an early stage [50]. Moreover, in *Epidendrum ibaguense*, similar small compact cells composed the SAM of protocorm [10]. The ability of PLB cells to divide rapidly makes them ideal explants for micropropagation and transformation studies. Despite the great advantage of PLB in large scale production and fundamental research, the regulating mechanism of PLB initiation and maintenance in *Paphiopedilum* remains unclear.

### The change of endogenous hormones levels on PLB development

The level of the major plant endogenous hormones IAA, GA, ABA, JA, and TZR were investigated during the four developmental stages of PLB. The results showed that the IAA level increased significantly during the latter two stages of PLB development. The accumulation of IAA might contribute to shoots emergence from PLBs. In *Cattleya tigrine*, a similarly significant rise of endogenous IAA content occurred when PLBs differentiated into shoots [53]. Besides the absolute contents, the transportation and distribution of auxin are crucial for PLB formation and shoot development [25]. Uniform auxin distribution helps maintain a dedifferentiated state until auxin is transported to specific locations to stimulate differentiation [54]. Polar auxin transport, mediated by PIN and AUX/LAX proteins, regulates the auxin distribution. The increased expression of four *PaphPINs* at stage B suggested that PINs-mediated auxin transport affects the development of *P.* SCBG Huihuang90 PLBs. Polar auxin transport analogously affected protocorm development in other orchids, such as *Spathoglottis plicata*. Lindleyana [26]. The function of *LAXs* during PLBs had not been studied. According to the expression pattern of two differentially expressed *LAX* in this study, it can be speculated that *PaphLAX1* was primarily involved in PLBs development, while *PaphLAX5* was primarily involved in shoots formation. Auxin early response genes, such as *AUX/IAA*, *GH3*, *TIR1,* and *ARF*, were reported to regulate shoot initiation and leaf development [55, 56]. Although the specific regulatory role has not been determined, these genes were potential candidates for PLBs developmental regulators. Further functional studies on the metabolism and signal transduction of IAA can be performed to identify their specific roles in PLB development.

The content of GA decreased significantly at the early stage of PLB development, which was consistent with the up-regulation of *PaphGA2*_*OX*_*1*. Previous studies showed that GA2ox can deactivate bioactive GAs and were expressed at the base of the SAM, restricting the influx of bioactive GA to regulate meristem function in several species [40, 44, 47]. GA is incompatible with meristem activities, and a low GA level is necessary for SAM activity [44]. JA content increased significantly with the differentiation of shoot from PLB. This was consistent with the conclusion that exogenous application of JA stimulated the PLB and shoot formation in *hybrid Cymbidium* [45]. The upregulation of the JA synthesis genes *LOX2*, *AOC*, *AOS1,* and *CYP74A2* may contribute to the increase in JA level.

### PLB initiation of *Paphiopedilum* may be a unique process, distinct from somatic embryogenesis

In recent years, the biological nature of PLBs has been controversial. PLBs are ideal explants for micropropagation because they have regeneration potential. Organogenesis and somatic embryogenesis are both morphogenetic processes leading to plantlet regeneration. PLBs are generally considered “somatic embryos” of the orchids because of their similarities in morphology and cell wall composition [8, 9, 57, 58]. To investigate whether PLB development of *Paphiopedilum* follows the somatic embryogenesis program, the expression patterns of the classic embryo markers including *SERK*, *BBM*, *CLV*, and *WUS* were examined. These embryonic markers are generally enriched in zygotic and somatic embryos, and their functions have been demonstrated to regulate the development of embryonic fate [17–19]. As shown in Supplemental Table S4, the expression level of *PaphSERK, PaphBBM1*, *PaphCLV,* and *PaphWUS* were either minimally detectable or remained consistent during the whole PLB developmental process. This shows that these genes were not suitable as marker genes for PLBs and that the molecular mechanisms of *Paphiopedilum* PLBs formation and somatic embryogenesis are different. However, *PaphBBM2* is an exception, whose function is important for cell proliferation and morphogenesis [59]. This conclusion is consistent with a recent study in *Phalaenopsis aphrodite*. Comparative transcriptome analysis of *Phalaenopsis aphrodite* showed that protocorm/PLB and somatic embryo shared few similarities in terms of gene expression profiles [16]. In addition, somatic tissues of *Phalaenopsis aphrodite* are considered morphologically different from PLBs [60]. Based on these results, it was proposed that PLB development of *Phalaenopsis aphrodite* is unique to shoot organogenesis instead of embryogenesis.

On the other hand, the initiation of cell division and establishment of auxin maxima are commonly observed at the early stage of de novo organogenesis [61, 62]. The endogenous IAA level increased, and the IAA biosynthesis, transportation, and signaling transduction genes were differentially expressed. In addition, the endogenous GA level decreased, which is consistent with previous reports that GA inactivation is required for the SAM initiation [63]. Taken together, PLB initiation of *Paphiopedilum* may be a unique process combining the characteristics of both organogenesis and somatic embryogenesis.

### Expression of *PaphWOX8* may be associated with PLB initiation

Since PLB development in *Paphiopedilum* may not follow the embryogenesis program similar to the model plants, searching for the genes that contribute to PLB initiation and development is important. Among the differentially expressed transcription factors (TF), the class TF *PaphWOX8* and *PaphWOX11* were particularly interesting because their expression level was correlated with the initiation of PLB and declined as the shoot formation started (Supplemental Table S4). Several members of the *WOX* family have been found to redundantly promote cell proliferation and prevent premature differentiation in meristematic tissues [64, 65]. In *Arabidopsis*, *AtWOX2*, *AtWOX8*, and *AtWOX9* can regulate asymmetric embryo lineage development [66]. Some of the *WOX* family genes can be activated by auxin during de novo organogenesis in herbaceous organisms [67, 68]. Functional characterization of these genes may help in identifying gene regulatory networks unique to *Paphiopedilum* PLBs.

## Conclusion

The transcriptome and endogenous hormone profile of *P.* SCBG Huihuang90 PLBs are reported here for the first time. The results revealed that a complex molecular regulatory network coordinates the induction and development of PLBs. Potential candidate genes involved in PLBs development are summarized. A variety of endogenous hormones co-regulate the development of PLBs. The histological characteristics of PLBs indicate that the cells of PLBs demonstrate a structure polarity. This study further supports the understanding and mechanism of PLBs initiation and development.

## Methods

### Plant materials

The *Paphiopedilum* species used in this study were hybridized by our lab and named *P*. SCBG Huihuang90. The seed parent was *P*. SCBG Prince, which was registered in The International Orchid Register (http://apps.rhs.org.uk/horticulturaldatabase/orchidregister/orchiddetails.asp?ID=972703) on 21 Feb, 2014. The pollen parent is *P*. SCBG Miracle, which was registered in The International Orchid Register (http://apps.rhs.org.uk/horticulturaldatabase/orchidregister/orchiddetails.asp?ID=963533) on 8 Mar, 2013. Both the seed parent and pollen parent were planted in a greenhouse in the South China Botanical Garden, Guangzhou, China and were cultivated under natural light with an average temperature and relative humidity ranging from 8 ~ 32 °C and 70 ~ 98%, respectively. After flowering and being artificially pollinated, mature seed capsules were collected for follow-up experiments.

### Seed germination and PLB induction

Capsules of *P*. SCBG Huihuang90 were washed with 70% alcohol three times and treated with 0.10% mercuric chloride for 20 min. After washing with sterile water three times, capsules were cut, and seeds were sowed in a seed germination medium made of Hyponex No. 26 medium [69] supplemented with 0.5 g/L activated carbon and 1.0 mg/L NAA. After 3 months, protocorms formed and were transferred to half-strength MS medium (Murashige and Skoog 1962) [70], which contained half-strength macro- and micro-elements of MS salts supplemented with 0.05 mg/L 2,4-dichlorophenoxyacetic acid (2,4-D) to induce the meristem mass. Meristem mass formed after 2 months and were transferred to the half-strength MS medium supplemented with 0.05 mg/L 2,4-D to induce PLBs. Subculture was conducted every 4 weeks. PLBs formed after 2 months and developed into different growth stages. PLBs were subcultured into the same medium every 2 months. Differentiated PLBs were transferred to PRG-free half-strength MS medium and cluster shoots formed after approximately 2 months.

The pH of all mediums was adjusted to 5.8 before autoclaving at 121 °C and 104 kPa for 15 min. All plant materials were cultured at 25 ± 1 °C and 12/12 h (light/dark) photoperiod of 40–45 μmol m^− 2^ s^− 1^ under cool white fluorescent tubes. Protocorm explants were cultured in a 9 cm glass flask with 100 mL solid medium for follow-up experiments.

### Sample collection

For phytohormone quantification, transcriptome sequencing, and quantitative real-time PCR, plant materials from different growth stages were collected, frozen in liquid nitrogen, and stored in a refrigerator at − 80 °C. Samples from each growth stage were taken at the same time and consisted of three biological replicates. The meristem mass, which had not formed obvious globular protuberance, formed after 2,4-D induction for 2 months. The materials of stage A were the green inner part of the mass of meristem. The materials of stage B were the mass of PLBs. The materials of stage C were the partly differentiated PLBs mass, which contained a mixture of shoots and PLBs. The materials of stage A-C were cultured on half-strength MS medium supplemented with 0.05 mg/L 2,4-D. The materials of stage D were the cluster shoots cultured on PRG-free half-strength MS medium. The weights of the samples were based on their experimental requirements.

#### PLB morphological characterization

##### Light microscopy

The plant materials from the four growth stages (described above) were observed and photographed under a stereo microscope (Nikon, SMZ745T, Japan) to study the morphological characters.

##### Paraffin section

The samples from the four growth stages (described above) were fixed in formalin acetic acid-alcohol solution (FAA; 70% ethyl alcohol: glacial acetic acid: 37% formaldehyde; 18:1:1) for a week. Fixed samples were dehydrated in a series of alcohols (70, 85, 95, 100, and 100%; v/v) for 1 h. Then, the samples were immersed in an ethanol-xylene mixture (2:1, 1:1, and 1:2; v/v) for 1 h, and xylene solution for 2 h. Paraffin was added to the solution until saturated at 36 °C overnight. Next, the temperature was gradually raised to 58 °C, and xylene was replaced by paraffin. Finally, the materials were embedded by paraplast after soaking in pure paraffin for 3 h. The wax blocks were sliced into 8 μm segments with a microtome (KEDEE, China). The dye used was Ehrlich’s haematoxylin (Biosharp, China), 1% safranin O (Solarbio, China), and 0.5% fast green dyes (Solarbio, China). The sections were observed and photographed under a biological microscope (Nikon, E200, Japan).

##### Phytohormone quantification

The determination of plant hormones was performed by high performance liquid chromatography-tandem mass spectrometry (HPLC-MS/MS). Tissue culture materials from each of the four growth stages were taken as experimental materials. Plant samples (50 mg) were ground into a powder in liquid nitrogen and added to 10 times the volume of acetonitrile solution. The material was extracted overnight at − 4 °C. After centrifugation at 12,000×g for 5 min, the supernatant was collected. To extract the hormone, 5 times the volume of acetonitrile solution was added to the precipitate, and the supernatant was collected and merged with the previous supernatant. After adding 25 mg CNW C18 QuEChers packing, the mixture was vortexed vigorously for 30s and centrifuged at 10,000×g for 5 min. The supernatant was collected and dried with nitrogen stream. The residues were redissolved in 200 μL methyl alcohol and filtered through 0.22 μm organic phase membrane. The mass spectrometer used is the Qtrap6500 (Agilent, America).

The results of the samples in each growth stage were represented by the average value of three biological replicates. The treatment data were subjected to analysis of variance (ANOVA) using IBM SPSS Statistics version 20. Duncan’s multiple range test at *P* value < 0.05 was performed with stage A as the control.

##### RNA extraction

Column Plant RNAout2.0 (Tiandz Inc., Beijing, China) was used for total RNA extraction performed according to the manufacturer’s instructions. Approximately 100 mg of each sample was used to extract RNA. The RNA was assessed using agarose gel electrophoresis, Nanodrop One (Nanodrop Technologies Inc., DE, USA), and Agilent 2100 (Agilent Technologies Inc., CA, USA) to confirm the purity, concentration, and integrity, respectively. The 260/280 nm ratios and 260/230 nm ratios of 1.8–2.2 and 1.6–2.2, respectively, from the Nanodrop were regarded as pure. Next, the RNA library was constructed, and sequencing was performed by Genepioneer Technologies Corporation (Nanjing, China). HiSeq4000 platform (Illumina Inc.) was used for high-throughput sequencing with a read length of PE150.

##### De novo assembly and functional annotation of unigenes

Transcriptome sequencing was performed on the samples from the four growth stages (described above), with three biological replicates for each stage. Raw data produced by sequencing were processed by removing adapters as well as filtering low quality reads with over 10% high unknown base (N) reads to obtain high quality clean data. Phred quality score Q20 and Q30 and GC-content of clean reads were calculated. Clean reads were assembled to finally obtain the unigene library of *P.* SCBG Huihuang90. The quality of transcriptome sequencing libraries was evaluated from three different perspectives: (1) examining the distribution of inserted fragments on unigene to evaluate the randomness of mRNA fragmentation and the degradation of mRNA; (2) drawing the length distribution map of the inserted fragment to evaluate the dispersion degree of the length of the inserted fragment; (3) evaluating whether the library capacity and the mapped reads compared to the unigene library were sufficient by drawing saturation map. All sequence data were uploaded into the BioProject database hosted by the National Center for Biotechnology Information (NCBI) under the BioProject PRJNA684752. Software BLAST (http://blast.ncbi.nlm.nih.gov/Blast.cgi) was used for functional annotations by comparing the information of sequence or amino acid sequence of unigenes to 7 databases: NR, Swiss-Prot, KEGG, COG, KOG, GO, and Pfam.

##### Unigenes expression and DEGs analysis

Bowtie (http://bowtie-bio.sourceforge.net/index.shtml) was used to compare the sequenced reads with unigene library. Fragments per kilobase per million mapped reads (FPKM) value was used to estimate the expression abundance of unigene. DEGs between libraries were identified by DESeq2 (http://www.bioconductor.org/packages/release/bioc/html/DESeq.html). Fold change represents the ratio of expression quantity between two samples, and the Benjamini-Hochberg approach was used to adjust the *P* values for controlling the FDR. Unigenes with FDR < 0.05 and an absolute value log2 (Fold change) ≥ 1 were considered differentially expressed. KEGG enrichment of DEGs was measured by enrichment factor, q value, and the number of genes enriched in the corresponding pathway.

##### Verification of gene expression using qRT-PCR

To validate the results of RNA-seq data, quantitative real-time PCR analysis was used to detect the expression levels of the 12 candidate genes. Primers were designed based on the Primer-BLAST [71] and listed in Supplemental Table S6. *ACT2* (TRINITY_DN57670_c1_g1) was selected as the reference gene for the normalization of the data. RNA of each growth stage was reverse transcribed using the One-Step gDNA Removal and cDNA Synthesis SuperMix kit (Trans, Beijing, China) according to the instruction manual, and cDNA of approximately 600 ng/μL was obtained. cDNA was diluted three times for subsequent experiments. 1 μL cDNA mixed with 0.8 μL primer pair (10 μM), 10 μL 2× Green qPCR SuperMix (Trans, Beijing, China), and 8.2 μL ddH2O. The mixture was used to carry out qRT-PCR detection on LightCycler 480 System (Roche Diagnostics, Germany). The amplification program was performed as follows: 30 s at 94 °C for initial denaturation, 40 cycles of 5 s at 94 °C for denaturation, 15 s at 57 °C for annealing, 10 s at 72 °C for elongation, followed by melting curve program for melting curve analysis. Each sample was processed as three biological replicates and three technical replicates. The relative expression was calculated by the 2^-ΔΔCt^ method [72].

## Supplementary Information

**Additional file 1: Supplemental Table S1**. Overview of de novo assembly. **Supplemental Table S2**. Annotated genes related to IAA metabolism and signaling. **Supplemental Table S3**. DEGs involved in jasmonic acid, brassinosteroid, ethylene, zeatin, salicylic acid and abscisic acid metabolism and signaling transduction. **Supplemental Table S4**. Annotated genes related to somatic embryogenesis. **Supplemental Table S5**. DEGs involved in photosynthesis. **Supplemental Table S6**. Primers used in the reference genes selection.

## Data Availability

All data generated or analyzed during this study are included in this published article and the supplementary information files. The sequence data was deposited in the NCBI database under the BioProject PRJNA684752.

## Abbreviations

PLBs: Protocorm-like bodies
*P.* SCBG Huihuang90: *Paphiopedilum*. SCBG Huihuang90
IAA: Indoleacetic acid
JA: Jasmonic acid
GA: Gibberellic acid
SAM: Shoot apical meristem
DEGs: Differentially expressed genes
CITES: International Trade in Endangered Species of Wild Fauna and Flora
ABA: Abscisic acid
HRGPs: Hydroxyproline-rich glycoproteins
TZR: Trans-zeatin
COG: Clusters of Orthologous Groups
GO: Gene Ontology
KEGG: Kyoto Encyclopedia of Genes and Genomes
KOG: euKaryotic Orthologous Groups
Pfam: Protein family
FDR: False discovery rate
PSII: Photosystem B
PSI: Photosystem
LHC: Light-harvesting complex
Cyt b_6_f: Cytochrome b_6_f
ATP: Adenosine triphosphate
qRT-PCR: Quantitative real-time polymerase chain reaction
TFs: Transcription factors
FAA: Formalin acetic acid-alcohol solution
HPLC-MS/MS: High performance liquid chromatography-tandem mass spectrometry
NCBI: National Center for Biotechnology Information
FPKM: Fragments per kilobase per million mapped reads
ANOVA: Analysis of variance
